# Supplementary material for: In Vitro and In Silico Antidiabetic Efficacy of Solanum lasiocarpum Dunal Fruit Extract
Source: PLoS One. 2025 Mar 10;20(3):e0312600. doi: 10.1371/journal.pone.0312600 (PMC11892833; doi:10.1371/journal.pone.0312600)
Supplement: Attachment — Submitted filename: RESPONSE TO REVIEWERS.docx [file pone.0312600.s002.docx]

**RESPONSE TO REVIEWERS’/EDITORS’ COMMENTS**

**Manuscript number:**

**Title:** ***In Vitro and In Silico Antidiabetic Efficacy of Solanum lasiocarpum Dunal Fruit Extract***

**Editor:**

| No. | Reviewers’/Editors’ comments | Response/Changes | Page no |
| --- | --- | --- | --- |
| 1. | In your work, you didn't tell us the relevance of running In-vitro first before in-silico. Before now, we have in-vitro and in-vivo. The in-silico was suppose to give relevant information needed to proceed to in-vitro. I understand that you may have your reasons for running in-vitro first, please state them and its relevance in the study. | Thank you for your comments:  For several reasons, running in-vitro experiments before in-silico simulations is crucial in microbiology and biomedical research. In-vitro studies validate hypotheses, provide tangible results, and offer a realistic environment to study biological interactions, which in-silico models may not fully capture. They generate empirical data for calibrating in-silico models, ensure accuracy, and provide insights into biological mechanisms like active compound interactions. Additionally, regulatory bodies often require in-vitro data before in-vivo studies, and practical constraints usually favor starting with in-vitro assays. | Methods |
| 2. | In your in-silico, and discussion, we didn't mention anything about comparison to a reference compound. In in-silico studies, it is essential to use reference drugs known to be active against the protein target of interest for comparison. | Thank you for your valuable feedback. We acknowledge the importance of comparing our findings to reference compounds from previous studies that are active against the protein target of interest. In our revised manuscript for discussion, we will incorporate reference drugs to serve as benchmarks for earlier studies. This comparison will allow us to validate our computational models and provide a more precise context for the efficacy and potential of the new compounds. | Discussion |
| Reviewer 1 | | | |
| 1. | Compare your approach with previous approaches. | Thank you for your comments. Our approach combines the strengths of in-vitro and in-silico methods, ensuring that the computational models are more accurately aligned with biological realities and enhancing the overall reliability of the research outcomes. | Discussion |
| 2. | Proofread the entire manuscript | Done: Thank you for your suggestion. | Manuscript |
| 3. | Draw a graphical abstract | Done: | Manuscript |
| 4. | Explain the novelty of the proposed approach. | Done: The novelty of our approach lies in conducting in-vitro experiments before in-silico simulations, enhancing model accuracy by grounding computational predictions in empirical data. This iterative process refines simulations with real-world insights, provides a comprehensive understanding of compound interactions, and identifies discrepancies early. This method ensures more reliable predictions and a more efficient drug development pipeline, offering a more accurate and practical approach than traditional methodologies. Our research findings from both laboratory experiments and computer simulations suggest that *S. lasiocarpum* has beneficial effects in treating diabetes. Consequently, it can be considered a viable supplementary formulation for preventing and treating diabetes. | Conclusion |
| **Reviewer 2** | | | |
| 1. | Please add the plant part used in the study (fruit) to the title. For example: In Vitro and In Silico Antidiabetic Efficacy of Solanum lasiocarpum Dunal Fruit Extract. | Done: In Vitro and In Silico Antidiabetic Efficacy of *Solanum lasiocarpum Dunal* Fruit Extract | Title |
| 2. | Please write out in full the species name (*Solanum lasiocarpum Dunal*) when it first introduced, then in the rest of the text the abbreviation could be used (*S. lasiocarpum*). | Done: thank you for your comments. | Abstract |
| 3. | Line 35: Could you please explain what you mean by mixed extracts from S. lasiocarpum? | Done: Apologies for the typo error. Mixed word removed | Abstract |
| 4. | to strengthen the introduction chapter, consider adding some literature or scientific reports that address the dose or dose range of S. lasiocarpum that have effects as traditional medicine. | Done: A paragraph was added to the introduction | Introduction |
| 5. | Although the chapter is well written, but in the Assessment of DPPH Radical Scavenging Activity Section (line 140), the authors stated ‘‘different volume sample extracts were made’’, however, these samples were not specified (what these samples represent, and how many doses were tested?). | Done: Different volume samples of *S. lasiocarpum* aqueous extracts were made in the range (0 to 5 mg/ml). | Method |
| 6. | In the Result chapter (lines 273-275), the authors said ‘‘With an IC50 of 10.2 ± 0.11 mg/mL, S. lasiocarpum demonstrated DPPH scavenging activity, however it was not as effective as Trolox, which had an IC50 of 0.69 ± 0.14 mg/mL. But in the Conclusion section (line 472-473), they said ‘‘The in vitro assessments showed that S. lasiocarpum had a higher antioxidant activity, as demonstrated by its ability to scavenge DPPH radicals’’. In light of these, the significance of this finding was not clearly explained, and it is well if you re-write theses sentences in an appropriate manner. | Done: Apologies for the technical error; the sentence was revised in conclusion (The *in vitro* assessments showed that *S. lasiocarpum* had a low antioxidant activity compared to trolox, as demonstrated by its ability to scavenge DPPH radicals) | Conclusion |
| 7. | A paragraph discussing the significance of some highlighted findings such as phenolic contents, DPPH radical scavenging activity, and cytotoxic effects with regard to the study objectives should be added to the discussion section. | Done: The significant phenolic content in *S. lasiocarpum* fruit extract underscores its potential as a rich source of natural antioxidants, aligning with the study’s objective of exploring antidiabetic bioactive compounds. The extract's high DPPH radical scavenging activity reflects its ability to neutralize free radicals, which is crucial for mitigating oxidative stress associated with diabetes (Santos et al., 2018). Additionally, the extract’s selective cytotoxicity towards certain cancer cell lines suggests its potential for developing complementary therapies for managing diabetes-related complications (Khan et al., 2020). These findings collectively support the therapeutic promise of S. lasiocarpum, highlighting its multifaceted biological activities in line with the study’s aims (Patel et al., 2019). | Discussion |
| 8. | The discussion section should be improved by relating or comparing the results of your study to previous related studies and/or potential future directions for research. | Done: the discussion improved and recommendations for future studies were added (These findings suggest that S. lasiocarpum could offer therapeutic benefits beyond diabetes management, potentially addressing cancer-related complications as well. Future research should focus on isolating specific phenolic compounds to better understand their individual contributions to antioxidant and cytotoxic activities. Additionally, exploring in vivo models and clinical trials could further validate the efficacy and safety of *S. lasiocarpum* for broader therapeutic applications, including its role in managing diabetes and related conditions. | Discussion |
| 9. | The in-text citation and references must be standardized according to the requirements of the journal. | Done: The text citations were revised. | Manuscript |
| 10. | Check the text carefully to eliminate some stylistic errors and ensure being standardized according to the requirements of the journal (line 76–84, renaming the Results & Discussion chapter to be Results or remove the Discussion chapter title and merge it with the previous one). | Done: The text was carefully checked and revised. The chapters are renamed to the result chapter and the discussion chapter. | Results |
| 11. | Authors frequently employed abbreviations throughout the manuscript without providing full introductions or explanations beforehand. | Done: the list of abbreviations was added after the list of references. | Manuscript |
